# Learning from the pandemic: Building capacity for risk communication in the Canadian federal health portfolio

**DOI:** 10.17269/s41997-025-01002-y

**Published:** 2025-03-14

**Authors:** Gabriela Capurro, Josh Greenberg

**Affiliations:** 1 Communications and Public Affairs Branch, Health Canada, Ottawa, ON Canada; 2 School of Journalism and Communication, Carleton University, Ottawa, ON Canada

**Keywords:** Risk communication, Public health, Capacity building, Training, Communication des risques, Santé publique, Renforcement des capacités, Formation

## Abstract

**Setting:**

The federal health portfolio has had a risk communications framework in place since 2006; however, the COVID-19 pandemic pushed the capacity of this plan and the need for communications resources to new levels. Health communicators in the public service face significant challenges: a fragmented mediascape, changes to how people seek and use information, the proliferation of misinformation and disinformation, declining trust in public institutions, and the politicization of science, to name just a few. It has never been more important for health authorities to communicate clearly, consistently, effectively, and from an evidence-based position.

**Intervention:**

This report describes one aspect of how the federal health portfolio has been addressing these challenges. As part of a recent capacity-building initiative, 67 public servants working in health communications participated in a four-part, half-day, advanced seminar series at Carleton University in June 2023. Each session featured an interactive presentation from a leading scholar and/or local practitioner with real-world scenario exercises designed to put their learning into practice. The series explored issues in trust and transparency, algorithmic control and mis- and disinformation, media relations, and risk communication for equity-deserving populations.

**Outcomes:**

At the conclusion of the program, participants were given tools to (1) identify challenges to effective communication brought by a rapidly evolving media environment in which skepticism and misinformation often run rampant; (2) examine how key metrics and behavioural indicators on social media platforms demand different responses from health organizations and agencies who are monitoring and managing social media; (3) consider challenges for health communicators who must serve the public during health crises while also reinforcing public trust in their institutions; and (4) develop successful risk communication strategies for equity-deserving communities by considering specific information needs and tailored dissemination methods to reach the intended audience. Participants expressed high levels of satisfaction in the quality of the training and overwhelmingly reported that it would positively impact their daily work.

**Implications:**

The training program was an innovative and successful initiative to improve knowledge of current priority topics and best practices in risk communication. It illustrated the benefits of continued professional learning, the importance of university-public service partnerships, and how capacity building requires ongoing resource commitments and engaged support from senior management. The program, along with other risk communication training that is currently being implemented, is part of the investment in long-term professional development of risk communicators in the health portfolio.

## Résumé

**Contexte:**

Le portefeuille fédéral de la santé dispose d’un cadre de communication sur les risques depuis 2006; cependant, la pandémie de COVID-19 a mis à l’épreuve la capacité de ce plan et la nécessité de ressources en communication à un niveau inédit. Les communicateurs en santé du secteur public font face à des défis importants: un paysage médiatique fragmenté, des changements dans la manière dont les gens recherchent et utilisent l’information, la prolifération de la désinformation et de la mésinformation, la diminution de la confiance dans les institutions publiques et la politisation de la science, pour ne citer que quelques exemples. Il n’a jamais été aussi important pour les autorités sanitaires de communiquer de manière claire, cohérente, efficace et sur une base factuelle.

**Intervention:**

Ce rapport décrit un aspect de la manière dont le portefeuille fédéral de la santé aborde ces défis. Dans le cadre d’une initiative récente de renforcement des capacités, 67 fonctionnaires travaillant dans la communication en santé ont participé à une série de séminaires avancés en quatre parties, d’une demi-journée chacun, à l’Université Carleton en juin 2023. Chaque session comportait une présentation interactive d’un universitaire de premier plan et/ou d’un praticien local, accompagnée d’exercices pratiques basés sur des scénarios réels conçus pour mettre en pratique leurs apprentissages. La série a abordé des questions liées à la confiance et à la transparence, au contrôle algorithmique et à la mésinformation et désinformation, aux relations avec les médias, ainsi qu’à la communication des risques pour les populations méritant de l’équité.

**Résultats:**

À la fin du programme, les participants ont acquis des outils pour (1) identifier les défis de la communication efficace posés par un environnement médiatique en évolution rapide où le scepticisme et la désinformation sont souvent omniprésents; (2) examiner comment les principaux indicateurs et mesures comportementales sur les plateformes de médias sociaux exigent des réponses différentes de la part des organisations et agences de santé qui surveillent et gèrent les médias sociaux; (3) considérer les défis auxquels sont confrontés les communicateurs en santé qui doivent servir le public en période de crise sanitaire tout en renforçant la confiance du public dans leurs institutions; et (4) développer des stratégies efficaces de communication des risques pour les communautés méritant de l’équité, en tenant compte des besoins spécifiques en matière d’information et des méthodes de diffusion adaptées pour atteindre le public cible. Les participants ont exprimé un haut niveau de satisfaction quant à la qualité de la formation et ont déclaré en grande majorité qu’elle aurait un impact positif sur leur travail quotidien.

**Conséquences:**

Le programme de formation a été une initiative innovante et réussie visant à améliorer la connaissance des sujets prioritaires actuels et des meilleures pratiques en matière de communication des risques. Il a illustré les avantages de l’apprentissage professionnel continu, l’importance des partenariats entre les universités et les services publics, et la manière dont le renforcement des capacités nécessite un engagement continu en ressources et un soutien actif de la part de la direction. Le programme, ainsi que d’autres formations sur la communication des risques actuellement mises en œuvre, fait partie de l’investissement dans le développement professionnel à long terme des communicateurs en santé.

## Introduction

In the last decade, health communicators have encountered major challenges due to changes in how people seek information and how they use it, the circulation of conspiracy theories, misinformation and disinformation, loss of trust in government and experts, and the politicization of science (Van der Meer, 2020; Thomas et al., 2022; Hart et al., 2020; Motta, 2020; Vieten, 2020). These challenges have been further deepened since the beginning of the COVID-19 pandemic, which brought scientific uncertainty, widespread anxiety, and the need for health authorities to communicate clearly, consistently, and effectively, sharply into view (Capurro et al., 2021; Greenberg & Everts, 2020). The federal health portfolio—comprising the Public Health Agency of Canada (PHAC), Health Canada (HC), and the Canadian Food Inspection Agency (CFIA)—has had a risk communications framework in place since 2006 that was tested and used through outbreaks of listeriosis, H1N1 influenza, and the COVID-19 pandemic, the last of which pushed the capacity of this plan and the health portfolio’s communications resources to new levels.

Since 2022, the federal health portfolio has been addressing these challenges by building evidence-based risk communication capacity. This effort involves internal work and collaborations with academia, including replacing the *2006 Federal Health Risk Communications Framework* with two new documents: the *2023 Research Synthesis Report on Health Risk Communication* and the *2024 Toolkit for Health Risk Communicators*, and the development of online self-paced training modules in risk communications. In this report, we describe another initiative for capacity building: the Risk Communication Summer Seminar Series.

This event was organized in collaboration with one of the authors, who is a leading Canadian scholar in risk communication, and was funded by Health Canada and the Canadian Food Inspection Agency. A total of 67 public servants working in communications in the health portfolio participated in four intensive half-day seminars at Carleton University in June 2023. The seminars were promoted for a month through internal newsletters and via emails, and participation was voluntary. Given the highly interactive design of the event, we limited it to 30 in-person participants and permitted 37 others to observe and ask questions online. Participants included communications advisors, managers, and directors. Most participants were female (83%). Each session featured an interactive presentation from a leading scholar and/or practitioner with real-world scenario exercises designed to put participants’ learning into practice. The series explored issues in trust and transparency, algorithmic control and mis- and disinformation, media relations, and risk communication with and for disenfranchised populations.

## Engaging public servants with current evidence and best practices

A first step was conducting a review of current scholarship and white papers published between 2012 and 2022. This literature review allowed us to map current evidence and best practices in risk communication and, in consultation with leaders in the federal health portfolio, identify priority areas for risk communication practice. Based on those priority areas, we developed four advanced learning sessions, each tackling specific challenges in risk communication practice, featuring an expert speaker who was either a scholar or a local practitioner, or both. Speakers were chosen by the authors of this article, who are risk communications scholars and practitioners with extensive experience and contacts in academia, industry, and government. Expert speakers assigned readings that participants were asked to complete before the seminar. Readings were provided in both English and French. Below we describe how each session was designed and their specific goals. Materials from the seminar sessions are available upon request, including the agenda, the scenarios, the speakers’ presentations, and the assigned readings.

### Session 1: Trust and transparency

This session introduced participants to the intricate web of public perceptions and opinions regarding trust in government and public institutions. The expert speaker, the CEO of a leading Canadian market research firm, explored with seminar participants the various sources, technologies, and platforms through which Canadians acquire information and their levels of trust and faith in “objective truths”, providing valuable insights for communicators navigating this complex and uncertain perception landscape. The session highlighted the challenges governments face when communicating publicly in a rapidly evolving environment in which skepticism and misinformation often run rampant. Participants were provided with current polling data and expert analysis, as they explored the complexities of modern communication and considered how best to foster trust in a world that is both deeply entwined and highly fragmented. Prior to their attendance at this session, participants reviewed the best practices report along with two industry reports, the *2023 Edelman Trust Barometer* (Edelman, 2023) and *The Crisis of Trust in Government and Democracy* (Proof Strategies, 2023).

### Session 2: Algorithmic control and mis-/disinformation

In this session, participants discussed the role of social media at a time when “information disorder” (Wardle, 2019) is posing significant risk to Canadians, particularly those who are drawn into social environments where false health narratives and conspiracies often collide with divisive political and social discourse. The seminar leader was a professor at Carleton University whose research expertise is in digital media and algorithmic culture. The participants together with the seminar leader explored some of the key issues around social media, algorithmic culture and control, and mis-/disinformation. Participants learned how key metrics and behavioural indicators on social media platforms, alongside particular kinds of content, demand different responses from organizations and agencies monitoring and managing social media. Some of the questions raised by the speaker included the following: What can we learn from social media account *activity* versus *visibility* in social spaces? What do these dimensions tell us about how algorithms control and shape conversations in the networked public sphere? What are some strategies for intervening on social media conversations that are amplifying mis- and disinformation? Before the session, participants reviewed *Understanding Information Disorder* (Wardle, 2019) and *“Suggested for You”: Understanding How Algorithmic Ranking Practices Affect Online Discourses and Assessing Proposed Alternatives* (Bundtzen, 2022). Participants who preferred French-language research were invited to review *Désinformation en période électorale: Comment la société civile peut-elle répondre?* (ISD, 2022) and *Les fausses nouvelles, nouveaux visages, nouveaux défis* (Sauvageau et al., 2018).

### Session 3: Media relations in a time of media fragmentation

This seminar was led by an experienced international health and science journalist and journalism professor, who discussed how the traditional role of government media relations has been upended in an era of the established business model of news media being in a state of collapse, the ascendance of social media in shaping everyday public information gathering, and turmoil in public trust of both governmental institutions and journalism, all against a backdrop of rising misinformation and disinformation. The seminar leader reflected on how the COVID-19 pandemic has deepened many challenges for health journalists and widened the choices for health communicators tasked with serving the public during an evolving health crisis while also reinforcing public trust in the institutions they serve. Participants discussed these challenges and choices, learned what journalists and journalism researchers are saying about the issues, and explored potential pathways for when the next public health crisis inevitably happens. The speaker assigned participants two readings: *The Shattered Mirror: News, Democracy and Trust in the Digital Age* (Public Policy Forum, 2017) (available both in English and French) and *Communicating Scientific Uncertainty in an age of COVID-19* (Fleerackers et al., 2022).

### Session 4: Risk communication with/for equity-deserving populations

In the last session, participants discussed how to ensure that risk messages reach historically disenfranchised, equity-deserving populations. The expert who led the seminar is a former journalist and currently the executive director of a non-profit organization that provides information and resources to immigrants and refugees in the national capital region. The seminar leader highlighted how immigrants, particularly refugees, have historically been inadequately prioritized in public health communication strategies. Therefore, it was no surprise to anyone working with newcomers that they would be at higher risk of infection during the COVID-19 pandemic. Yet, early pandemic risk communication strategies repeatedly failed to meet these populations’ needs. Risk messages were not sufficiently translated into their languages or dialects, not framed in culturally relevant terms, and not disseminated properly to reach them. The speaker explained that many risk communicators learned rapidly that they did not know enough about these vulnerable populations or their information needs. Drawing on lessons learned and exploring real-world examples, the session challenged participants to work with key building blocks for developing successful risk communication strategies for equity-deserving communities by considering their information needs, relevance of information, and adequate dissemination methods to reach the intended audience. Although the speaker did not assign any readings for this session, they did share two documents in advance explaining information-seeking behaviours across minority groups, and accessibility to health information as an equity issue.

### Scenario-based exercises and group discussions

After the expert presentation, the speaker and training leader introduced a scenario exercise to participants or questions for discussion which had been planned in advance (see Table 1). Participants had to discuss the best strategy for communicating a health risk in a specific situation related to the issue of the day. Participants were divided into small working groups either with members of their team or with colleagues from other departments and tasked to work together through a set of guiding questions. The purpose of these exercises was to challenge participants to put into practice what they had learned during the session, and to consider several operational and ethical issues that may arise in such situations. Participants were given 20–30 min for group discussions, and were given the option of working in a different, quieter space. After the group discussions, participants returned to the larger group and shared how their group proposed to communicate the risk presented in the scenario, including potential challenges and constraints they could face as risk communicators in the public service. Examples of these challenges were lengthy approval processes, lack of timely expert information, and not having enough capacity to engage with stakeholders in a meaningful way.

**Table 1:** Seminar topics and group discussion questions

Topic	Group discussion questions
Trust and transparency	Context: Trust in public institutions and officials has declined since the pandemic Problem: From Brampton, Ont. to sites in nearby Halton and Niagara regions, there are a growing number of reports of birds that have tested positive for highly pathogenic avian flu virus, or bird flu 1. How important is it now to publicly communicate all that is known about the potential risks of an avian influenza pandemic? 2. Is there a risk that public trust could be threatened by fully disclosing all that is known about the risks of an avian influenza pandemic? 3. Who should be the public face in communicating whatever is currently known about the risks of an avian influenza pandemic? 4. What strategic options does your organization face today in building public trust as you prepare for the possibility of an avian influenza pandemic?
Algorithmic control and mis-/disinformation	What are some strategies for intervening on social media conversations that are amplifying mis- and disinformation? 1. Bundtzen recommends a series of interventions that are more strategic to address content issues. How feasible are some of the ideas catalogued in their report? (see pp. 16–24) 2. Are there social media strategies that your organization can develop or has developed to counter disinformation?
Media relations in a time of media fragmentation	Scenario A: A new prion disease has emerged in meat in Canada. Nobody knows the exact origin, but it has clearly spread through the global meat industry. Until it is under control every meat product needs to be thoroughly cooked… to a crisp. Ultimately this means you need to develop a message that BBQ-ing is unsafe this summer. Please develop key messages needed to reach two important audiences, and develop a plan for delivering the key messages in both a standard press conference and a short video. Scenario B: The health impacts of vaping were sidelined during the pandemic for obvious capacity reasons. Despite being marketed as a fun and safe alternative to smoking, there’s a public health imperative to alert Canadians of the long-term risk of lung disease and the addictive potential of vaping. Please develop key messages needed to reach two important audiences, and develop a plan for delivering the key messages in both a standard press conference and a short video.
Risk communication for disenfranchised populations	Based on what you’ve heard today, please discuss the following questions in your groups 1. Which perceptions or practices identified in this presentation do you see or have you seen in your own organization that might be barriers to effective risk communication to newcomers and racialized audiences? Why are they used? 2. Which three actions do you think your organization or your team could take to begin to change your approach? 3. What are the main barriers to making those changes in your organization? How might you overcome them?

## Evaluation

The day after the last session, participants were sent an invitation via email to complete a voluntary evaluation survey. The survey was hosted on the online platform Microsoft Forms in both English and French, so that participants could fill it out in the official language of their choice. The survey comprised 14 questions for in-person participants and 13 for online participants. The questions asked about participants’ perceived level of knowledge before and after the event, the usefulness of what they learned in the seminars and scenario-based exercises, the quality of the lectures and speakers, the difficulty and usefulness of the readings, the venue, and the overall experience. Participants had 1 week to complete the survey.

A total of 34 participants (51%) completed the survey, most of whom rated the usefulness of the seminars as “excellent” (44%) or “very good” (44%), and as positively impacting their daily work (79%). An overwhelming majority of respondents stated that they would recommend the seminar series to a colleague (91%). Participants also had the opportunity to write additional comments on what other topics and pedagogical elements they would like to see in future risk communication trainings. One third of respondents left a comment, clearly communicating an interest in learning more about risk communications. Among the suggestions made in the survey were a deeper discussion of risk theory, more tabletop exercises, a clearer link between the content and how to apply it in public service, and a stronger focus on behaviour change.

## Outcomes

At the conclusion of the program, participants had the tools to (1) identify challenges to effective communication brought by a rapidly evolving media environment in which skepticism and misinformation often run rampant; (2) examine how key metrics and behavioural indicators on social media platforms demand different responses from health organizations and agencies monitoring and managing social media; (3) consider challenges for health communicators who must serve the public during health crises while also reinforcing public trust in their institutions; and (4) develop successful risk communication strategies for equity-seeking communities by considering specific information needs and tailored dissemination methods to reach the intended audience. Participants expressed high levels of satisfaction in the quality of the training and overwhelmingly reported that it would positively impact their daily work.

The Risk Communication Summer Seminar Series was an innovative and successful training initiative by the Federal Health Portfolio to learn from the challenges of the COVID-19 pandemic and build health risk communication capacity in the public service. The method employed for the seminar series is flexible enough that it can be applied to risk communication training for public health professionals more broadly, such as risk assessors and policy analysts, as well as in different levels of government (federal, provincial, territorial, municipal). Our participants were recruited based on their roles and communication responsibilities in the health portfolio, instead of their demographic characteristics. However, the seminar series addressed risk communications from an intersectional perspective by raising awareness and understanding of socioeconomic determinants of health and the unique health information needs of equity-seeking communities. Future training event organizers should try to recruit communicators from a diversity of backgrounds, and continue emphasizing the intersectional nature of health risks.

The seminar series was part of an investment strategy in long-term professional development of risk communicators in the federal health portfolio, which included replacing the *2006 Federal Health Risk Communications Framework* with two new documents: the *2023 Research Synthesis Report on Health Risk Communication* and the *2024 Toolkit for Health Risk Communicators*, as well as the development of online self-paced training modules in risk communications, launched in the fall of 2024. These interventions seek to infuse risk communication principles and best practices into the portfolio’s communication activities in order to improve the reach and effectiveness of potentially life-saving risk messages for people in Canada. This learning intervention allowed health communicators to learn from experts in the field, who challenged them to think creatively about how best practices would work in real-life situations. By the removal in the scenario exercise of the multiple layers of approvals that public health communicators face in their daily work, the participants were able to learn in an engaging and cooperative manner. Finally, hosting the seminar series in a nationally recognized research-intensive university was important to conveying the high standard of training of the seminar. Health portfolio organizations are science-based and they place a high value on the production of evidence-based communications; therefore, senior leadership was very supportive of the seminar series. This training was deemed valuable by health portfolio leadership because it offered specialized training that could be delivered in Ottawa, whereas other options for risk communications training are limited and often involve travel and accommodations.

## Implications for policy and practice

What are the innovations in this policy or program?

- While seminars are a staple for training in the public service, this intervention allowed public servants to (1) learn from leading experts in fields of direct relevance to their work, (2) critically review current evidence related to public health risk communication, and (3) apply both the evidence and lessons learned during the COVID-19 pandemic in tabletop exercises.
- Analyzing evidence and applying it to real-world scenarios, in addition to listening to and engaging in discussion with leading subject matter experts, is an innovative pedagogical approach that encourages evidence-based health risk communication practice.

What are the burning research questions for this innovation?

- The method employed for the seminar series is flexible enough that it can be applied to risk communication training for public health professionals more broadly, such as risk assessors and policy analysts, as well as in different levels of government (federal, provincial, territorial, municipal). However, some questions remain: Can the seminars be held with a larger group of participants or does the format work better in smaller groups? How can participants be recruited for equity and inclusion? How can support from senior leadership be secured for this type of learning intervention?

## Data Availability

Available upon request.
